# *In vivo* measurement of pediatric extracorporeal oxygenator insensible losses; a single center pilot study

**DOI:** 10.3389/fped.2024.1346096

**Published:** 2024-02-29

**Authors:** Tess L. Suttles, John Poe, Tara M. Neumayr, Ahmed S. Said

**Affiliations:** ^1^ Division of Critical Care Medicine, Department of Pediatrics, Washington University in St. Louis, St. Louis, MO, United States; ^2^ Mechanical Support Department, St. Louis Children's Hospital, St. Louis, MO, United States; ^3^ Institute of Informatics, Washington University in St. Louis, St. Louis, MO, United States

**Keywords:** extracorporeal membrane oxygenation, intensive care units, pediatric, insensible losses, fluid overload

## Abstract

**Introduction:**

Fluid overload on Extracorporeal Membrane Oxygenation (ECMO) is associated with worse outcomes. Previous *in vitro* studies have attempted to quantify oxygenator-related insensible losses, as failure to account for this fluid loss may lead to inaccurate fluid balance assessment and potentially harmful clinical management, such as unnecessary exposure to diuretics, slow continuous ultrafiltration (SCUF), or continuous kidney replacement therapy (CKRT). We performed a novel *in vivo* study to measure insensible fluid losses in pediatric ECMO patients.

**Methods:**

Pediatric ECMO patients were approached over eleven months in the pediatric and cardiac intensive care units. The water content of the oxygenator inflow sweep gas and exhaust gas were calculated by measuring the ambient temperature and relative humidity at frequent intervals and various sweep flow.

**Results and discussion:**

Nine subjects were enrolled, generating 431 data points. The cohort had a median age of 11 years IQR [0.83, 13], weight of 23.2 kg IQR [6.48, 44.28], and body surface area of 0.815 m^2^ IQR [0.315, 1.3725]. Overall, the cohort had a median sweep of 2.5 L/min [0.9, 4], ECMO flow of 3.975 L/m^2^/min [0.75, 4.51], and a set ECMO temperature of 37 degrees Celsius [36.6, 37.2]. The calculated net water loss per L/min of sweep was 75.93 ml/day, regardless of oxygenator size or patient weight. There was a significant difference in median documented vs. calculated fluid balance incorporating the insensible fluid loss, irrespective of oxygenator size (pediatric oxygenator: 7.001 ml/kg/day [−12.37, 28.59] vs. −6.11 ml/kg/day [−17.44, 13.01], respectively, *p *= 0.005 and adult oxygenator: 14.36 ml/kg/day [1.54, 25.77] and 9.204 ml/kg/day [−1.28, 22.05], respectively, *p* = <0.001). We present this pilot study of measured oxygenator-associated insensible fluid losses on ECMO. Our results are consistent with prior *in vitro* methods and provide the basis for future studies evaluating the impact of incorporating these fluid losses into patients' daily fluid balance on patient management and outcomes.

## 1 Introduction

Extracorporeal Membrane Oxygenation (ECMO) is a life-sustaining support modality for children and neonates with cardiac and/or respiratory failure. As ECMO outcomes have improved, ECMO use has increased worldwide (1). Fluid overload (FO) accumulated during the ECMO course has been associated with poor patient outcomes (2–5), including increased mortality, intensive care unit length of stay, and duration of mechanical ventilation (6, 7). Unfortunately, accurate measurement of fluid balance for patients on ECMO is difficult. Traditionally documented net fluid balance only accounts for easily measurable intake (enteral or parenteral) and output (urine, stool, and other quantifiable drainages). Insensible losses are not commonly accounted for and occur via water loss by diffusion through the skin, or by evaporation during respiration. ECMO oxygenator evaporative losses potentially add an additional source of insensible fluid losses (8–12). Not accounting for these additional insensible losses on ECMO, especially in younger patients, may impact clinical decision-making with overly aggressive measures to mitigate a positive fluid balance with diuretics, slow continuous ultrafiltration (SCUF), or continuous kidney replacement therapy (CKRT). Such measures are not without risk as there can be worsening of renal function, excessively negative ECMO circuit inlet pressures leading to subsequent hemolysis, and there is limited data regarding CKRT's impact on anticoagulation management while on ECMO (13–15).

To date, studies evaluating ECMO oxygenator-associated insensible water loss were all performed *in vitro* (8–12). The estimated insensible water losses had a wide range depending on the oxygenator, and none of the studies used blood-primed circuits, rather measuring water loss from clear liquid primed circuits (8–12). Additionally, these studies kept constant sweep gas flow rate, blood flow rate, and/or temperature during the experiments, as opposed to clinical scenarios when these variables change multiple times over the course of an ECMO run. In this study, we performed a direct measurement of the insensible oxygenator fluid losses *in vivo* from pediatric patients supported on ECMO. We aimed to establish a method to measure insensible water loss from the oxygenator for patients on ECMO and to investigate patient and ECMO factors associated with water loss.

## 2 Materials and methods

This study was approved by the Washington University in St. Louis Institutional Review Board (approval date 08/04/2021, number 202106001), and the devices used in the study met the FDA definition of non-significant risk. Eligible patients were approached and consented within six hours of ECMO initiation. As many patients in this study were minors and too critically ill to consent or assent, a parent or legal guardian gave informed consent. The procedures were followed in accordance with the ethical standards of the institutional committee on human experimentation and with the Helsinki Declaration of 1975.

We performed a single center, prospective study of all patients supported on ECMO (veno-arterial (V-A) or veno-venous (V-V)), in our pediatric intensive care unit (PICU) or cardiac intensive care unit (CICU) from January 2022 to November 2022. Neonatal intensive care unit ECMO patients were excluded due to the use of a different ECMO circuit configuration utilizing roller pumps. As per institutional practice, enrolled patients were supported using custom ECMO circuits composed of centrifugal pumps and oxygenators using custom 1/4- or 3/8-inch tubing from Medtronic with Cortiva BioActice Surface®. For patients less than 14 kg, a ¼ inch circuit was used incorporating an Affinity® centrifugal pump (Medtronic, Minneapolis, MN) with a Bioline-coated pediatric 2.8-L/min Quadrox-ID® (Getinge, Göteborg, Sweden) oxygenator. While for patients greater than 14 kg, circuits with custom 3/8 inch tubing were utilized including an Affinity centrifugal pump with an adult 7.0-L/min Biolined Quadrox-ID® oxygenator (Getinge, Göteborg, Sweden). All circuits incorporated a Cincinnati Sub-Zero ECMO Heater Model 333W (Cincinnati, OH.) Each patient had the circuit primed per institutional protocol and all clinical management decisions were made by the treating clinical team including systemic anticoagulation per the institutional hemostasis and thrombosis guidelines (16).

Demographic information, underlying pathology that led to ECMO deployment, and ECMO characteristics were obtained from the medical record. A REDCap database was created to store documented daily intake and output measurements, daily weight when available, and percent daily fluid overload calculated as daily fluid intake (L) minus daily fluid output (L) divided by admission weight (kg), multiplied by 100, hourly ECMO parameters, and multiple laboratory values. Laboratory values included: hemoglobin, hematocrit, platelet count, renal function, markers of coagulation including thromboelastography (TEG), and markers of hemolysis.

### 2.1 Insensible fluid loss measurements

**Figure 1:**
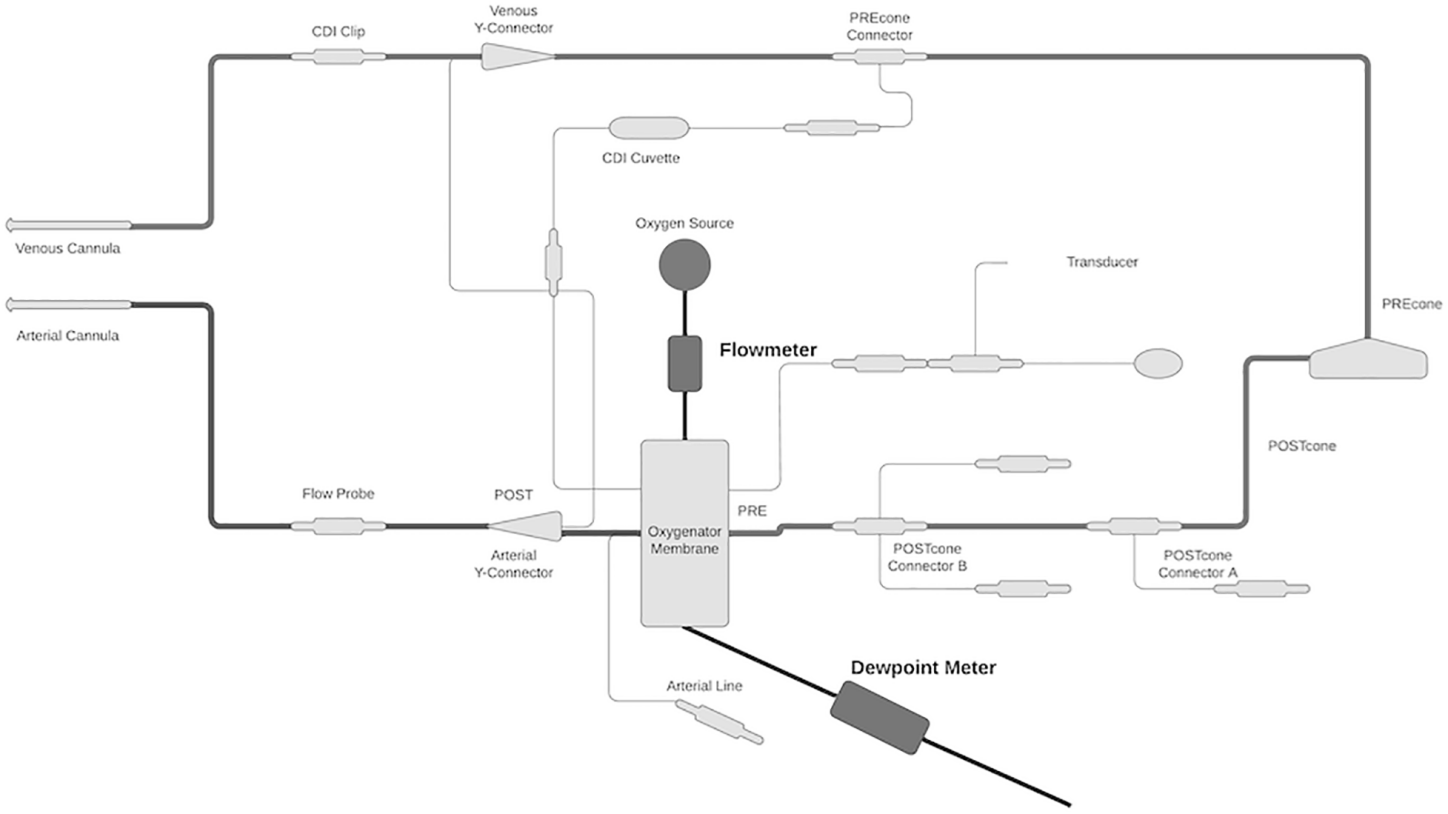
Schematic of study setup. The digital flowmeter was attached to the sweep inflow for an accurate measurement of sweep gas flow rate. The dew point meter was attached to the oxygenator exhaust port to measure relative humidity and ambient temperature. These measured values were used to calculate absolute water content.

To ascertain fluid loss from the oxygenator, a digital TSI flowmeter was attached to the sweep inflow for accurate oxygenator sweep measurements because previous studies have found sweep rate to be a major influencer of water loss. Standard ECMO circuits utilize a bubble flowmeter, so a digital flowmeter was used for more accurate sweep flow measurements. A DeFelsko dew point meter was affixed to the exhaust port of the oxygenator and measured the relative humidity of the exhaust gas and ambient temperature to calculate absolute humidity (Figure 1). Measurements were taken every 15 min and stored on a local computer without patient-identifying data.

Calculation of absolute humidity or water content was ascertained using the relative humidity and ambient temperature measured by the dew point meter. To accurately measure the water loss, the digital flow meter and dew point meter were used to measure the water content of both sweep inflow and the oxygenator exhaust gases. First, water vapor saturation pressure (*P*_ws_) was calculated, in hPA, with ambient temperature (*T*), in Celsius, Equation 1:

(1) $$P_{ws}=A\;\cdot \;10^{\left(\frac{m\;\cdot \;T}{T+T_{n}}\right)}$$

*A, m, and T_n_* are constants, 6.116441, 7.591386, and 240.7263, respectively (17).

Second, the vapor pressure (*P*_w_), in hPA, was calculated using the measured relative humidity (RH) and previously calculated *P*_ws_, Equation 2 (17):

(2) $$P_{w}=\frac{RH\cdot P_{ws}}{100}$$

Third, absolute humidity (AH), in g/m^3^, was calculated with *P*_w_, in hPA, and measured *T*, in Kelvin, of the exhaust gas, Equation 3.


(3) $$AH=C\;\cdot \;\frac{P_{w}\cdot 100}{T}$$

*C* is a constant, 2.16679 gK/J (17).

AH was calculated in g/m^3^ and then converted to ml/hour per L/min of sweep and then to ml/day per L/min of sweep.

### 2.2 Statistical analysis

Statistical analysis was performed using GraphPad Prism version 9. Descriptive statistics of the subjects and ECMO parameters were calculated, with categorical variables reported as numbers and percentages and continuous variables as median and interquartile range [IQR] unless otherwise stated. Mann–Whitney *U*tests and Wilcoxon Signed Rank tests were used to compare continuous variables and Fisher's exact tests were used for categorical variables. Linear regression was performed to assess the association of the calculated water loss with continuous ECMO variables. A two-tail *p*-value of less than 0.05 was used to signify statistical significance.

## 3 Results

Forty-one patients met criteria for enrollment during the study period. Twenty-four patients were excluded; one patient was Spanish-speaking only, one patient died prior to being approached, and twenty-two patients could not be approached within the enrollment deadline. Of the seventeen patients approached, twelve consented. Nine subjects were included in the final analysis; two were excluded as they were concurrently on CRRT in line with the ECMO circuit and one had data loss (Supplementary S1).

Of the entire cohort, five (55%) were male. The median age was 11 years IQR [0.83, 13] and the median weight was 23.2 kg IQR [6.48, 44.275]. Median body surface area (BSA) was 0.815 m^2^ IQR [0.315, 1.3725]. The leading indication for ECMO initiation was cardiogenic shock (44%), and the leading configuration was V-A, five subjects (55%). Four subjects (45%) were supported with a pediatric oxygenator, and five subjects (55%) were supported with an adult oxygenator. Subjects supported on circuits with an adult oxygenator had statistically significantly higher ECMO blood flow, sweep gas flow rate, cardiac index, and ECMO set temperature (0.57 L/min vs. 4.28 L/min, *p* = <0.0001, 0.9 L/min vs. 3.5 L/min, *p* = <0.0001, 2.043 L/m^2^ vs. 2.645 L/m^2^, *p* = <0.0001, and 37.4°C vs. 36.6°C, *p* = <0.0001, respectively). Table 1 shows patient and ECMO characteristics for the entire cohort and then compared by oxygenator size (Pediatric vs. Adult Quadrox).

**Table 1:** Study subjects and ECMO characteristics.

Variable Median [IQR]	All subjects *n* = 9	Pediatric Oxygenator *n* = 4	Adult Oxygenator *n* = 5	*p*-value
Male, *n* (%)	5 (55)	3 (60)	2 (40)	0.523
Age, years	11 [0.83–13]	0.54 [0.187–0.872]	13 [12–14]	0.015
Weight, kg	23.2 [6.48–44.27]	7.24 [5.22–8.92]	45.7 [40–45.9]	0.015
Height, cm	108.25 [56.62–156.37]	60.75 [51.87–70.12]	158 [151.5–16]	0.015
BSA, m^2^	0.81 [0.315–1.37]	0.35 [0.26–0.42]	1.39 [1.32–1.43]	0.015
Weight at ECMO initiation, kg	23.55 [5.32–44.97]	6.45 [4.12–9.05]	45.3 [44–48.3]	0.015
Peak %FO	4.8 [0.55–9.85]	7.2 [3.425–13.62]	1.3 [0.3–4.3]	0.555
ECMO indication				
Cardiogenic shock, *n* (%)	4 (44)	2 (50)	2 (40)	0.522
Acute hypercarbic RF, *n* (%)	1 (11.5)	1 (25)	0 (0)	
Acute hypoxemic RF, *n* (%)	3 (33)	1 (25)	2 (40)	
Septic shock, *n* (%)	1 (11.5)	0 (0)	1 (20)	
ECMO configuration				
V-V, *n* (%)	4 (45)	2 (50)	2 (40)	>0.999
V-A, *n* (%)	5 (55)	2 (50)	3 (60)	
Cannulation modality				
Peripheral, *n* (%)	6 (67)	2 (50)	4 (80)	0.523
Central, *n* (%)	3 (33)	2 (50)	1 (20)	
ECMO duration, hours	288 [211.27–434.78]	220 [148.92–281.78]	434.8 [288–1,345.05]	0.111
Flow, L/min	3.975 [0.75–4.5]	0.57 [0.44–0.8]	4.28 [4.02–5.02]	<0.0001
Sweep, L/min	2.5 [0.9–4]	0.9 [0.7–1]	3.5 [2.8–4]	<0.0001
Set temperature, °C	37 [36.6–37.2]	37.4 [37.2–37.4]	36.8 [36.5–37]	<0.0001
Cardiac index, L/min/m^2^	2.55 [2.04–2.73]	2.04 [1.64–2.21]	2.645 [2.53–3.03]	<0.0001
Net water loss, ml/day per L/min of sweep	75.93 [29.25–75.93]	75.93 [23.24–75.93]	75.93 [30.07–75.93]	0.199
ICU survival				
Yes, *n* (%)	5 (55)	3 (75)	2 (40)	0.523
No, *n* (%)	4 (45)	1 (25)	3 (60)	
ICU length of stay, days	36 [27.75–48.5]	29 [27.75–36]	43 [36–46]	0.674

BSA, body surface area; FO, fluid overload; ECMO, extracorporeal membrane oxygenation; RF, respiratory failure; V-V, veno-venous; V-A, veno-arterial; ICU, Intensive care unit.

*p*-value by Chi Square or Fisher Exact test.

Subject characteristics for the entire cohort, and then separated by Pediatric Oxygenator and Adult Oxygenator.

**Figure 2:**
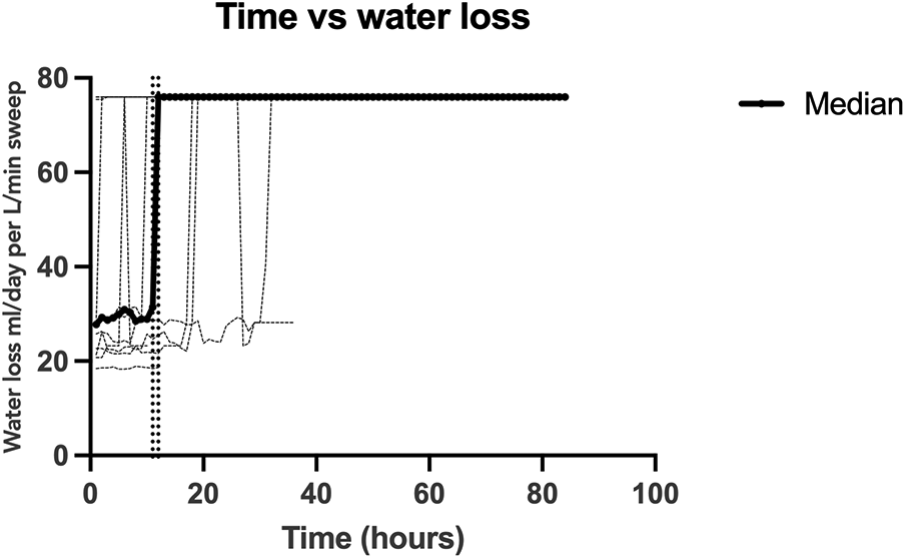
Calculated oxygenator-associated water loss over time. A depiction of the calculated water loss over time for each individual data collection session (light gray) and the median for the studied cohort (black). The two vertical dotted lines show the inflection point when water loss reached a steady equilibrium, between 11 and 12 h. All subjects reached this equilibrium of 76 ml/day/l/min of sweep which was also the median of the cohort.

Data was collected over a total of twelve sessions, with 431 data points over 108 h. The absolute humidity and water content was calculated for the sweep inflow and exhaust to derive a net fluid loss. The peak ambient temperature of the exhaust gas was 42.5°C (range 20.3°C–42.5°C) and the peak relative humidity was 99.4% (range 93.7%–99.4%). After reaching this peak, all subjects remained at equilibrium with a constant water loss. The sweep inflow ambient temperature was 24.1°C and the relative humidity was 21.8%, which resulted in an absolute water content of 6.86 ml/day/L/min of sweep. For the entire cohort, the net median water loss was 75.93 ml/day/L/min of sweep IQR [31.07, 75.93]. Figure 2 depicts the measured water loss over time per individual subject and the median for the whole cohort. Each subject reached the peak water loss which coincided with the median water loss of the entire cohort. All subjects reached this peak water loss within 48 h, with the median water loss reaching a peak at 18 h IQR [6, 28] (Supplementary S2).

**Figure 3:**
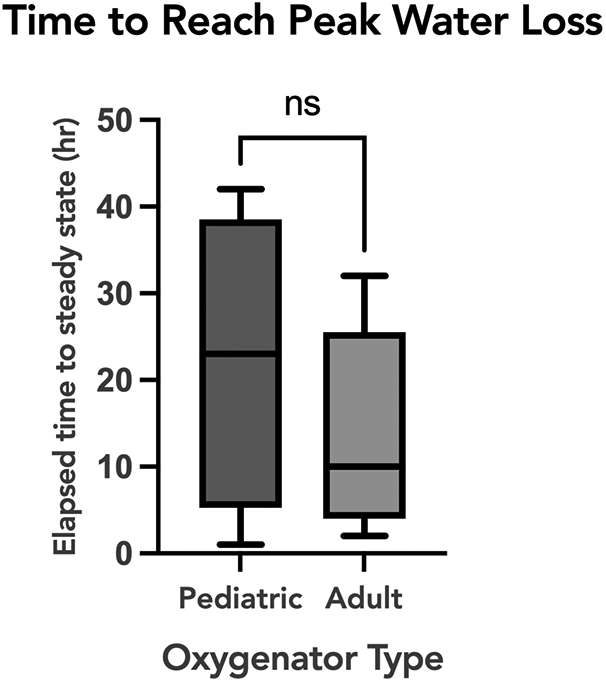
A comparison of time to reach a steady equilibrium for calculated water loss based on oxygenator size. Box and whisker plot of the time to reach equilibrium for peak calculated water loss between the two oxygenator sizes. The median time to reach peak water loss for the pediatric oxygenator was 23 h IQR [13.75−31.5] and for the adult oxygenator was 10 h IQR [6−19], *p* = 0.73.

**Figure 4:**
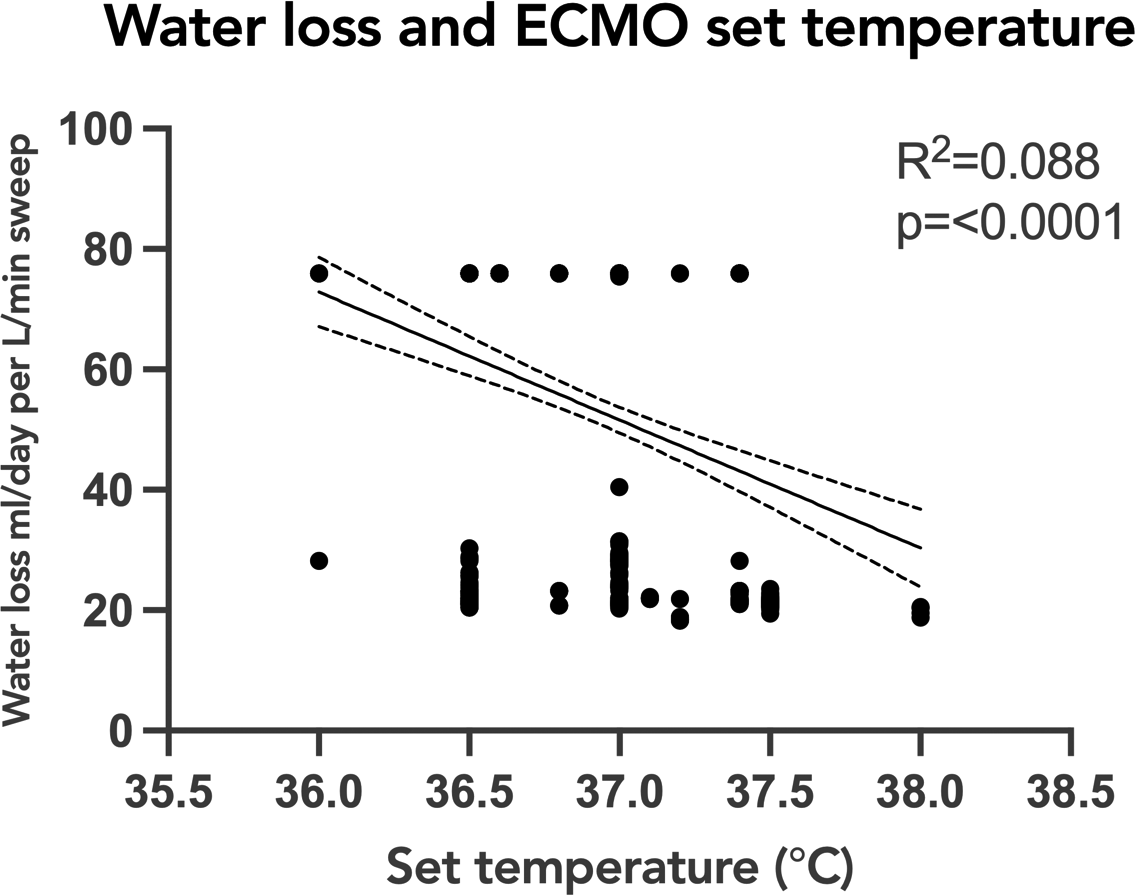
Linear regression analysis of calculated oxygenator water loss with ECMO set circuit temperature. Simple linear regression depicting the relationship of oxygenator-associated water loss to ECMO circuit set temperature. There was a statistically significant association between set ECMO circuit set temperature and calculated oxygenator water loss, *p*=<0.0001, with an R^2^ value of 0.088. The correlation was also weak and the trend was decreased water loss with higher set temperatures. ECMO, extracorporeal membrane oxygenation.

**Figure 5:**
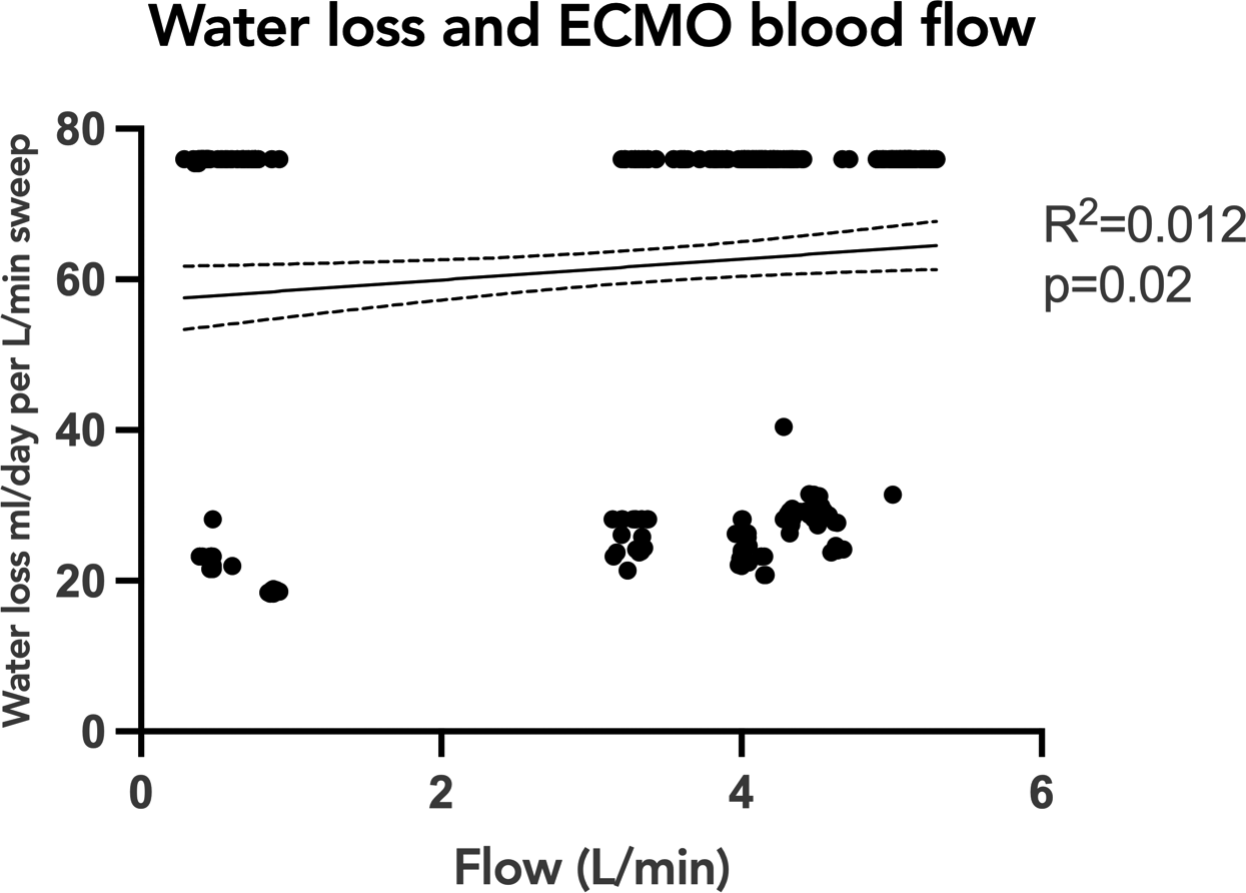
Linear regression analysis of calculated oxygenator water loss with ECMO blood flow. Simple linear regression analysis showing the relationship of calculated oxygenator-associated water loss to ECMO blood flow. There was a statistically significant association between ECMO blood flow and calculated oxygenator water loss, *p *= 0.02, with an R^2^ value of 0.012. Although significant, the correlation was weak. ECMO, extracorporeal membrane oxygenation.

To evaluate the association of oxygenator water loss with various subject and ECMO circuit variables, we assessed the association with oxygenator type, ECMO blood flow, and set temperature on the ECMO circuit. Sweep is taken into account with the water loss calculation, so was not included in the comparison. When comparing the time to reach peak water loss based on oxygenator type, there was no statistically significant difference between subjects supported on Pediatric vs. Adult Quadrox-ID oxygenators, *p *= 0.7302. The median time to reach peak water loss for the pediatric oxygenator was 23 h IQR [13.75, 31.5] and for the adult oxygenator was 10 h IQR [6, 19] (Figure 3). Notably, the net difference in water lost by oxygenator size was not statistically significant, with pediatric oxygenator median 75.93 ml/day/L/min of sweep IQR [75.93, 75.93] and adult oxygenator median 75.93 ml/day/L/min of sweep IQR [30.07, 75.93], *p *= 0.4939. On linear regression analysis, there was a statistically significant association between ECMO circuit set temperature and calculated oxygenator water loss, *p* = <0.0001, with an *R*^2^ value of 0.088 (Figure 4). Similarly, there was a statistically significant association between blood flow and calculated oxygenator water loss, *p *= 0.02, with an *R*^2^ value of 0.012 (Figure 5).

**Figure 6:**
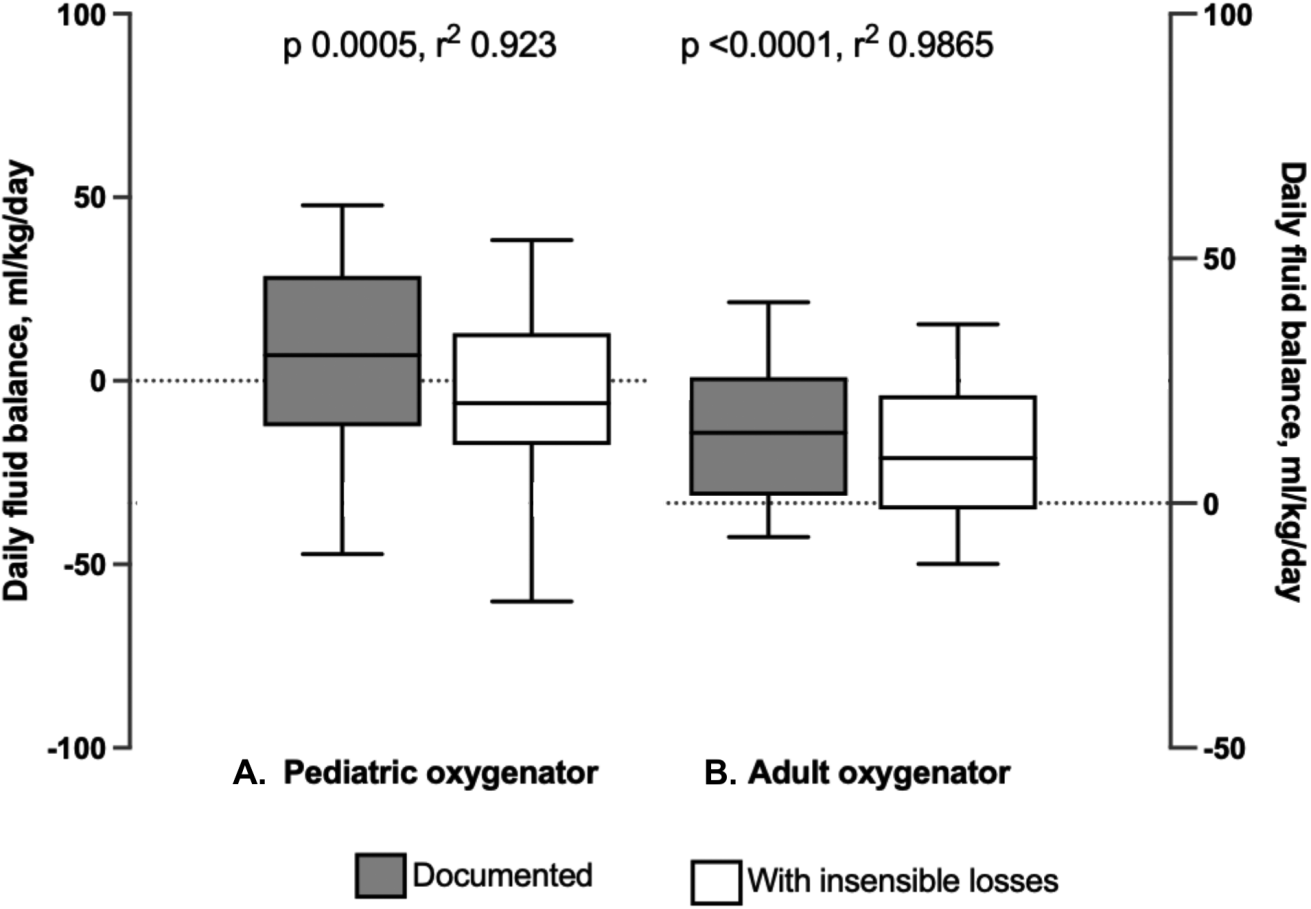
Comparison of daily fluid balance for both pediatric and adult oxygenators, with and without insensible losses. (**A**) Box and whisker plots of fluid balance in ml/kg/day as documented compared to fluid balance with insensible losses included in patients supported on the Pediatric Oxygenator. There was a significant difference, *p* = 0.005. (**B**) Box and whisker plot of fluid balance as documented compared to fluid balance with insensible losses included in patients supported on the Adult Oxygenator. There was a significant difference, *p* < 0.001.

Finally, documented fluid balance was compared with fluid balance including calculated insensible loss, by multiplying hourly sweep flow rate and measured hourly water loss from the oxygenator. The fluid balance per kilogram of body weight was compared across the pediatric and adult oxygenators. For the pediatric oxygenator cohort, there was a significant difference when comparing the documented fluid balance per kg body weight and fluid balance including the calculated insensible losses per kg body weight (7.001 ml/kg/day IQR [−12.37, 28.59] and −6.11 ml/kg/day IQR [−17.44, 13.01], respectively, *p* = 0.005) (Figure 6A). Similarly, for patients supported on the adult oxygenator, there was a significant difference, comparing the documented fluid balance per kg body weight and fluid balance accounting for the calculated insensible losses per kg body weight (14.36 ml/kg/day IQR [1.54, 25.77] and 9.204 ml/kg/day IQR [−1.28, 22.05], respectively, *p* = <0.001) (Figure 6B). Additionally, the percent change in body weight over the course of the ECMO run utilizing the documented and calculated fluid balance including oxygenator insensible losses, were a median of 8.3% IQR [7.5, 13.7] and 7.9% IQR [6.7, 11.6] for pediatric oxygenator subjects and adult oxygenator subjects, respectively.

## 4 Discussion

A cumulative positive fluid balance on ECMO has been associated with poor outcomes. Accurate assessment of fluid loss is hindered by the inability to account for insensible fluid losses, including oxygenator-associated water loss, which may subject patients to unnecessary and potentially harmful attempts to counteract fluid overload. Possible negative effects of excessive fluid removal—either by administration of diuretics or use of SCUF and/or CKRT—may include increased hemolysis, reduced cardiac support via lower delivered ECMO flow, and thereby exacerbation or instigation of acute kidney injury. Additionally, it is still unclear the effects of CKRT use on anticoagulation parameters, further complicating ECMO management. In this single-center prospective *in vivo* pilot study, we found that oxygenator-associated insensible water loss through the ECMO circuit was primarily dependent on sweep gas flow rate, at a rate of approximately 76 ml/day per L/min of sweep.

Prior assessments of oxygenator-associated water loss have been limited to *in vitro* studies utilizing clear liquid primed circuits. The method presented in this study for measuring oxygenator-associated water loss demonstrates a substantial amount of fluid loss, a median of 190 ml/day for the studied cohort. This degree of water loss if added to daily fluid balance totals has the potential to influence clinical management, especially in small pediatric patients.

Our *in vivo* findings are consistent with previous *in vitro* studies demonstrating that sweep gas was the major driver of water loss in ECMO circuits (8–12). Measured insensible water loss via various oxygenators in these studies ranged from 48 ml/day per L/min of sweep to 83 ml/day per L/min of sweep; our study demonstrated oxygenator-associated water loss that is at the upper end of this range (8–12). Camacho et al. studied the Avecor oxygenator and designed a study to compare oxygenator size, blood flow rates, and sweep in relation to insensible water loss. They studied two sizes of oxygenators of the same brand and measured water loss at varying ECMO blood flow and sweep gas flow rates. The water loss was measured after exhaust gas had condensed in a suction canister and via a replacement method whereby a burette added fluid to the circuit to account for the loss. The authors concluded that sweep gas flow rate was the only determinative factor for oxygenator water loss (9). Alexander et al. investigated *in vitro* water loss via the Medtronic Minimax Oxygenator by measuring direct water loss and changes in sodium concentration over time (8). The circuit flow was kept constant and the change in water loss and sodium concentration was found to be linear and dependent on the sweep gas flow rate (8). Gill and O'Shaughnessy demonstrated that heating the sweep gas made no difference in water loss, but that heating and humidifying the sweep gas significantly decreased water lost through the circuit (10).

In this study, we designed a novel method for *in vivo* measurement of insensible loss from the oxygenator from pediatric patients supported on ECMO. The method used to measure insensible loss in our study illustrated that the exhaust gas reached equilibrium after a median of 18 h as it became fully saturated with water. All patients reached equilibrium, despite variations in blood flow rate, temperature, and oxygenator size. Although there was no significant difference in time to reach equilibrium based on oxygenator size, there was a trend that the larger adult oxygenator came to equilibrium faster. This may have occurred due to the larger surface area in the adult oxygenators allowing for a greater gas-fluid interface to achieve equilibrium faster. More importantly, the size of the oxygenator did not affect the rate of insensible water loss.

As expected, there were significant differences in demographic data and ECMO indices based on oxygenator size. Younger, smaller patients received lower ECMO blood flow and sweep gas flow rates. Although the sweep requisite for these patients was less than for older, larger patients, accurate estimation of the amount of oxygenator insensible fluid losses may be more impactful for a small infant as compared to an older child or adolescent. This was demonstrated when comparing documented fluid balance with fluid balance including insensible losses. The pediatric oxygenator cohort (smaller patients) had a negative fluid balance per kg body weight when including insensible losses, but a positive documented fluid balance. If this water loss is not accounted for in the daily fluid balance, a patient may appear more fluid-positive than they actually are, and they may receive more diuretics with potential consequences or may be initiated on renal replacement therapy when it might not be warranted. However, even with including insensible losses in daily fluid balance, there was still an impressive increase in percent body weight change for both pediatric and adult oxygenator patients, highlighting the need for more accurate fluid balance measurements.

Unexpectedly, the correlations of water loss to both temperature and blood flow were statistically significant but had a low coefficient of determination, indicating a significant finding, but a small contribution by either temperature or blood flow. One previous study had suggested an association between the temperature of the fluid and the water loss (12). Our results showed a trend in the opposite direction: as the temperature of the fluid increased the trend line was weakly negative. It is important however to note that the range of temperatures set on the ECMO circuits in all the studied subjects was narrow, and only physiologic temperatures were used. The analyses produced two groupings of data points, with a smaller amount of water loss across a range of temperatures and then a larger amount of water loss across a different range of temperatures. An explanation for these findings could be that the two groupings of data points were a consequence of the water loss not yet reaching equilibrium. A future direction would be to investigate if higher set temperatures would correlate with a faster time to reach equilibrium. Similarly, our data demonstrated a significant but weak correlation between ECMO blood flow rate and calculated water loss, with two groupings of data points scattered across varying levels of flow. These findings are congruent with previous studies demonstrating that ECMO flow was not an important factor associated with the degree of water loss from the ECMO circuit (8–12).

Although this study included multiple data collection sessions and many data points, the small number of patients analyzed may have contributed to some of these unexpected findings and limited our results. Specifically, we recognize the difference in scale between documented and expected fluid balance in ml/kg/day and % change in body weight. While a larger sample size would likely produce more precise estimates of these differences, we feel that both represent clinically meaningful differences between documented and calculated fluid balances, and warrant further inquiry. The inclusion of the percentage helps to align our data with the recently published literature on fluid overload and fluid balance from a nomenclature perspective. Another limitation of this study was the loss of data for one of the subjects. Additionally, at times it was challenging to attach the Dew Point meter to the pediatric oxygenator as there are two ports for exhaust. This required splicing tubing together to allow for a single area of data collection. This study was also limited in that it was performed at a single center with identical circuit configurations except for the size of the oxygenator. It would be important to validate these findings in a larger study population with varying ECMO circuit configurations. Furthermore, it is still unknown if accounting for this insensible loss from the oxygenator in fluid balance could impact clinical management or patient outcomes.

In conclusion, patients on ECMO have potentially clinically significant insensible water loss via the ECMO oxygenator. This loss is constant after reaching an equilibrium and primarily correlates with sweep gas rates. Water loss is not dependent on the size of the oxygenator and is consistent across the spectrum/range of patient sizes. With physiologic temperature used on an *in vivo* ECMO circuit, the impact of temperature on insensible water loss was negligible. Further studies are necessary to confirm and validate this method for water loss measurement in other circuit configurations, and to evaluate the impact of incorporating oxygenator-associated insensible water losses in the overall fluid balance of ECMO patients on clinical management and patient outcomes, including exposure to diuretics or the use of adjuvant renal replacement therapies.

## Data Availability

The raw data supporting the conclusions of this article will be made available by the authors, without undue reservation.
