# Supplementary material for: In vivo measurement of pediatric extracorporeal oxygenator insensible losses; a single center pilot study
Source: Front Pediatr. 2024 Feb 29;12:1346096. doi: 10.3389/fped.2024.1346096 (PMC10937534; doi:10.3389/fped.2024.1346096)
Supplement: Supplementary file 1 [file Datasheet1.docx]

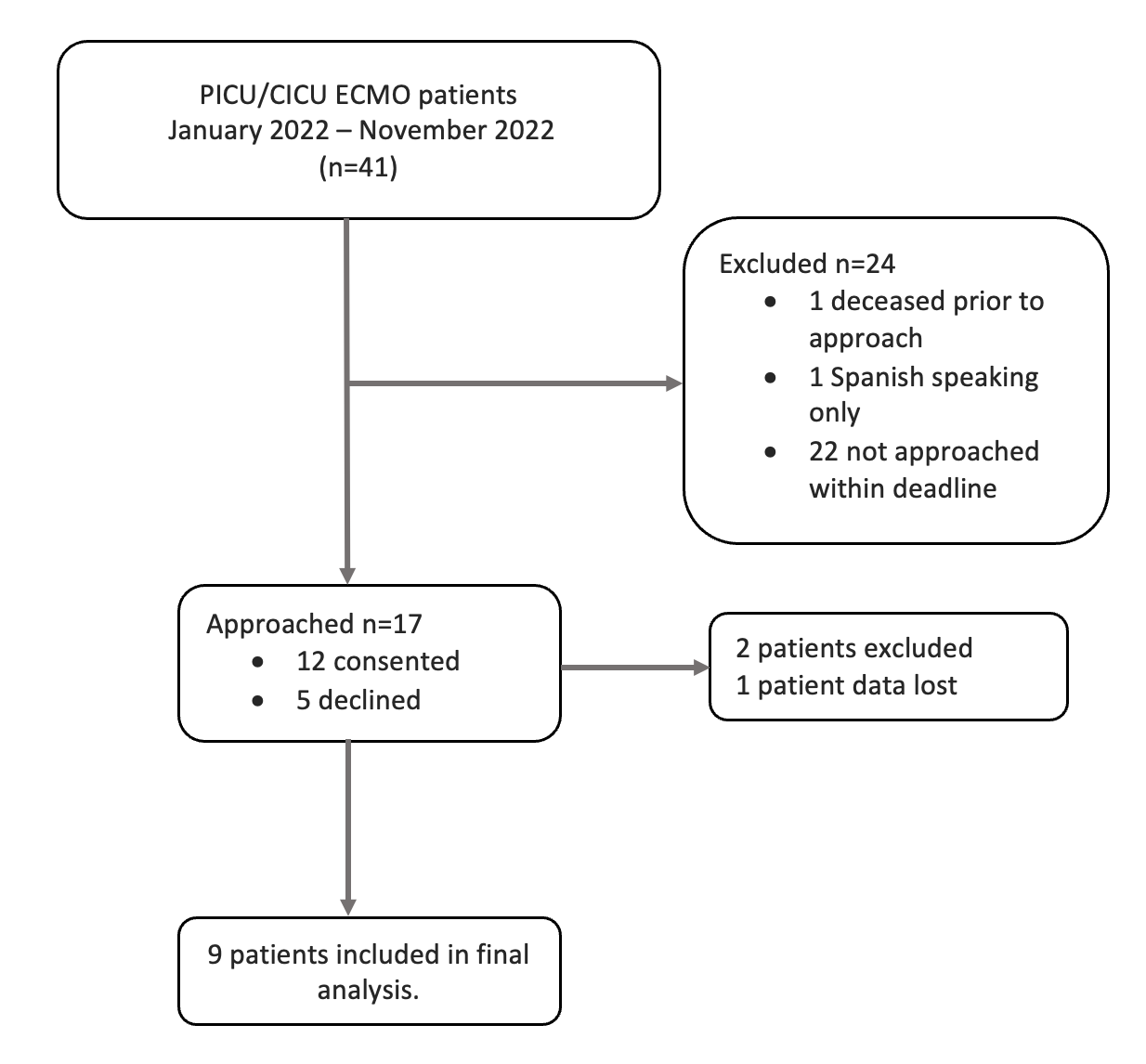


**Supplement 1. Patient selection and enrollment diagram.**

Pediatric Intensive Care Unit (PICU), Cardiac Intensive Care Unit (CICU), Extracorporeal Membrane Oxygenation (ECMO). During the study period, 41 patients received ECMO support. Twenty-four patients were excluded; one was Spanish speaking only, one died before the study team approached and 22 were not approachable in the study enrollment period. Seventeen patients were approached, twelve consented and five declined participation. Of the 12 patients who consented, two patients were excluded as they were concurrently on renal replacement therapy and one patient had data loss. Nine patients were included in the final analysis.


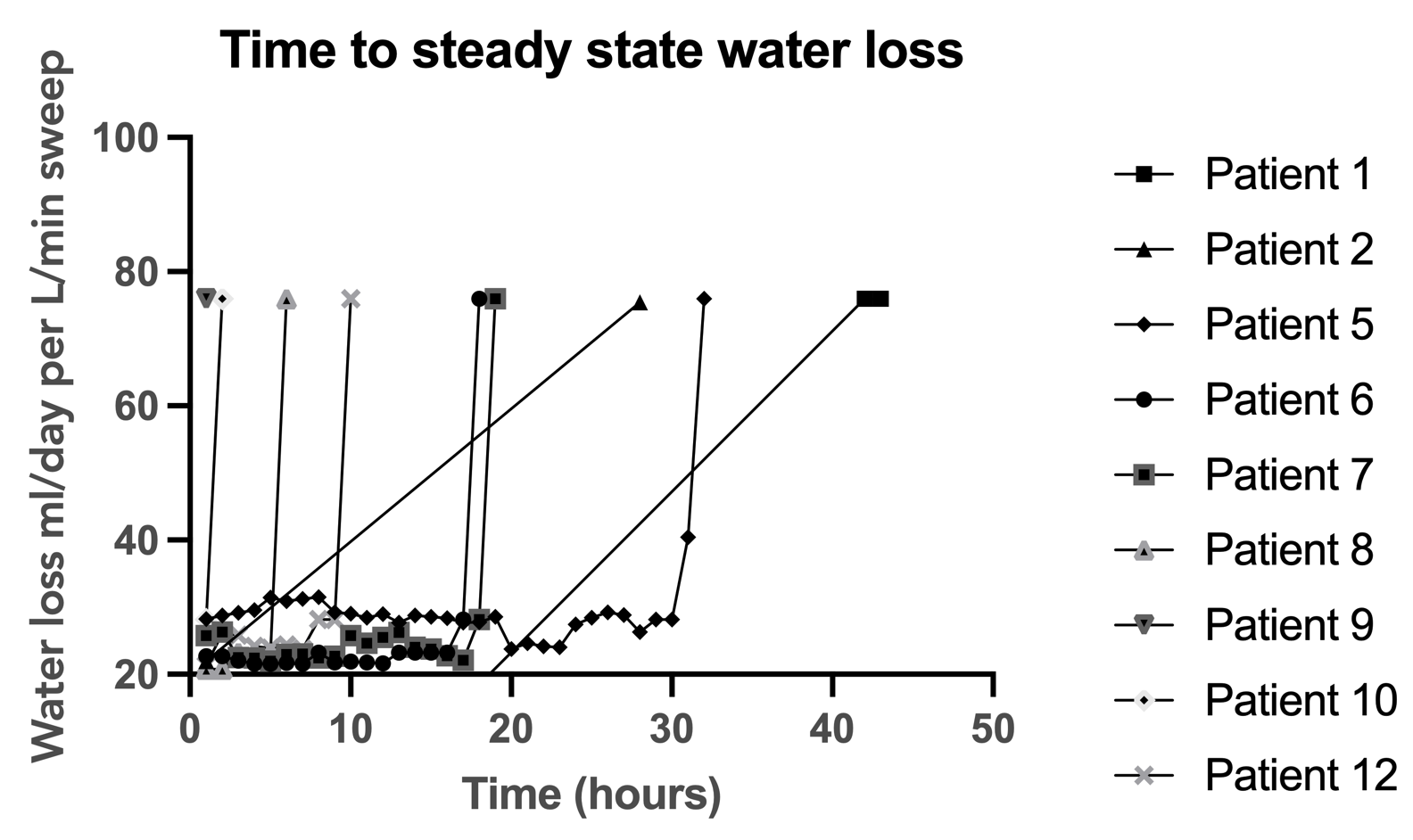


**Supplement 2. Calculated oxygenator water loss over time for each subject.**

This figure depicts water loss over time per subject. All enrolled subjects reached equilibrium within 48 hours.
